# Serum antibody levels to SARS-CoV-2 receptor-binding domain (RBD) in convalescent patients and vaccinated individuals of northern Nevada

**DOI:** 10.1371/journal.pone.0288713

**Published:** 2023-11-02

**Authors:** Derrick Hau, Kathryn J. Pflughoeft, Marcellene A. Gates-Hollingsworth, Simranjit Kaur, Haydon J. Hill, Jose Arias-Umana, Chelsea C. Chung, Valerie L. Smith, Mark S. Riddle, Sara A. Healy, David P. AuCoin

**Affiliations:** 1 Department of Microbiology and Immunology, University of Nevada, Reno School of Medicine, Reno, Nevada, United States of America; 2 Department of Internal Medicine, University of Nevada, Reno School of Medicine, Reno, Nevada, United States of America; 3 Renown Health, Reno, Nevada, United States of America; St Jude Children’s Research Hospital, UNITED STATES

## Abstract

Antibodies reactive with the SARS-CoV-2 receptor-binding domain (RBD) of the spike protein are associated with viral neutralization, however low antibody titers, specifically against SARS-CoV-2 variants, may result in reduced viral immunity post naturally acquired infection. A cohort study comprised of 121 convalescent individuals from northern Nevada was conducted looking at anti-RBD antibody levels by enzyme-linked immunosorbent assay. Serum was collected from volunteers by staff at the University of Nevada, Reno School of Medicine Clinical Research Center and assessed for antibodies reactive to various SARS-CoV-2 RBD domains relevant to the time of the study (2020–2021). A nonpaired group of vaccinated individuals were assessed in parallel. The goal of the study was to identify antibody levels against the RBD subunit in convalescent and vaccinated individuals from northern Nevada.

## Introduction

Coronavirus disease 2019 (COVID-19) is caused by severe acute respiratory syndrome coronavirus 2 (SARS-CoV-2), and has led to over 650 million cases reported globally [1]. Most individuals infected with the SARS-CoV-2 will elicit an antibody response against viral nucleocapsid (N) protein and spike (S) protein within 1–3 weeks post-symptom onset [2–4]. A subset of antibodies reactive with the receptor-binding domain (RBD) of the S protein have demonstrated neutralizing activity by blocking viral fusion to host cells, mediated by the interaction to human ACE2 receptor [4, 5]. Despite antibody-mediated neutralizing activity, further research is warranted to understand natural and vaccine-induced immunity against SARS-CoV-2 virus as breakthrough infections by variants have been identified in both convalescent and vaccinated populations [6, 7]. Titers against the RBD domain of dominant circulating variants, between 2020–2021, were assessed using samples collected from a cohort of individuals in northern Nevada that were naturally infected with SARS-CoV-2. Additionally, titers against Wu-Hu-1 RBD were assessed among individuals who received vaccines against SARS-CoV-2 and reported no history of COVID-19 disease.

## Materials and methods

The cohort for this study included 121 adults recruited from the general population in northern Nevada, that had recovered from acute SARS-CoV-2 infection, tested negative for at least two weeks, and were ≥18 years old at enrollment. A summary of patient characteristics, along with method of diagnosis (nucleic acid amplification tests (NAAT) or serodiagnosis), can be found in S1 Table. Enrollment occurred at two sites: Renown Health in Reno, Nevada and the University of Nevada, Reno School of Medicine Clinical Research Center (CRC) (UNR IRB#1590416–20). Written consent was obtained prior to sample collection. Serum samples were collected at the time of enrollment between May 2020 and February 2021. Additionally, serum samples from unmatched vaccinated volunteers, age of ≥18 years, were collected under a separate University of Nevada, Reno protocol (UNR IRB#1696183–5) between June and October 2021. Verbal consent, as authorized by the IRB, was obtained at time of sample collection. Vaccinated volunteers did not report a history of a known SARS-CoV-2 infection and had completed a full vaccine regimen of either BNT162b2 (Pfizer-BioNTech), mRNA-1273 (Moderna), or Ad26.COV2.S (Johnson & Johnson–Janssen).

**Fig 1:**
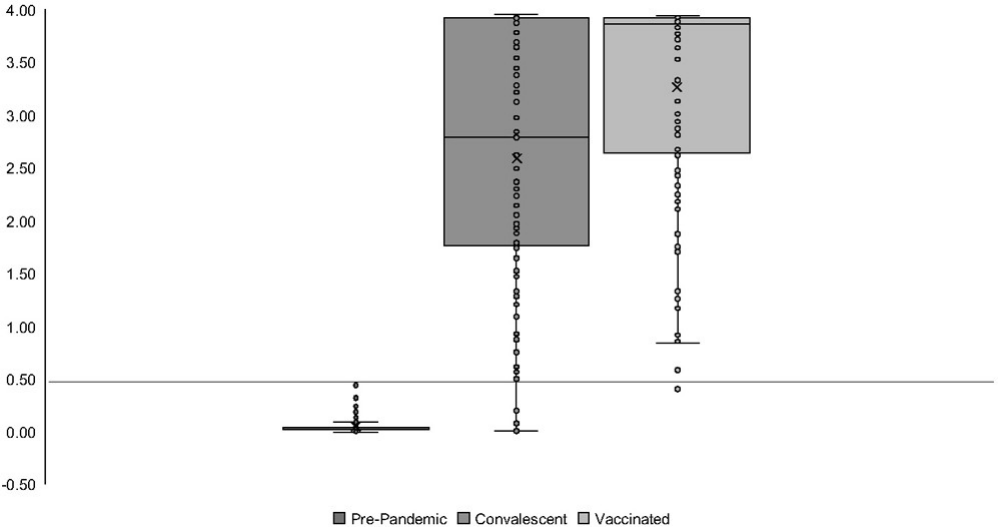
Serum samples collected from convalescent patients (n = 121; IQR: 59–118) and vaccinated volunteers (n = 101; IQR: 125–190) were evaluated for seropositivity by ELISA using a cutoff value of OD_450_ ≥ 0.5 at a dilution of 1:100 (indicated by line); a value established from archived pre-pandemic sera from 2007 (n = 152).

Serum was analyzed for immunoglobulin G (IgG) antibodies reactive against recombinant RBD sequences of the S protein from multiple variants of SARS-CoV-2 (GenScript, Piscataway, NJ): Wu-Hu-1 (Z03479), Alpha (Z03533), Beta (Z03613), Kappa (Z03607), Delta (Z03613) through an in-house enzyme-linked immunosorbent assay (ELISA) that was adapted from *Amanat*, *et al*. [8]. Briefly, serum was heat-inactivated at 56°C for 1 hour and stored at -80°C until use. Microtiter plates (Greiner Bio-One, cat. No. 655001) were coated with the RBD domain of the S protein (1 μg/mL) in 50mM carbonate buffer at 4°C overnight. All assay washes were performed on a microplate washer (Biotek ELx405, Winooski, VT) using 300μL of phosphate-buffered saline containing 0.5% Tween-20 (PBST). Assay microtiter plates were blocked with 300μL of 5% milk in PBST for 1 hour at 37°C. Serum samples were diluted into sample buffer (PBST containing 5% milk and 3M NaCl) at a 1:100 dilution. Endpoint titers were determined using two-fold serial dilutions between 1:100 and 1:12,800. Samples were incubated within microtiter wells (assay volume ‐ 100 μL) for 30 minutes at room temperature (RT). Plates were washed, then incubated with 100 μL of 0.04 μg/mL goat anti-human IgG HRP (Abcam, cat. No. ab97225) in 5% milk in PBST for 30 minutes at RT. Plates were then washed and binding was assessed by introducing 100 μL TMB substrate (Seracare, cat. No. 5120–0050) for 30 minutes at RT. An equal volume of 1M H_3_PO_4_ was used to stop the reaction, and colorimetric data was read at OD_450_. Pre-pandemic samples archived from 2007–2008 (n = 152) were used to establish an analytical OD_450_ cutoff of 0.5 resulting in 100% assay specificity at a 1:100 dilution (Fig 1). Seropositivity was defined as an OD_450_ ≥ 0.5 at a 1:100 dilution. Endpoint titers were established as the greatest dilution resulting in OD_450_ ≥ 0.5. The Mann-Whitney Rank Sum Test was used to assess the statistical difference in the median endpoint titers between groups. The Wilcoxon sum rank test was used to assess significance between titers to different variants.

## Results

Among naturally infected individuals, convalescent serum was collected on average 87 days (IQR: 58–118) post-symptom onset. A summary of patient characteristics and their serology results can be found in S1 Table. The average age of the enrolled cohort was 45 years old. Of the 121 individuals, 119 were confirmed positive for SARS-CoV-2 by either nucleic acid amplification tests (NAAT) (100/119) or serodiagnosis (19/119) between March-December 2020. Two individuals were suspected based on symptoms.

**Fig 2:**
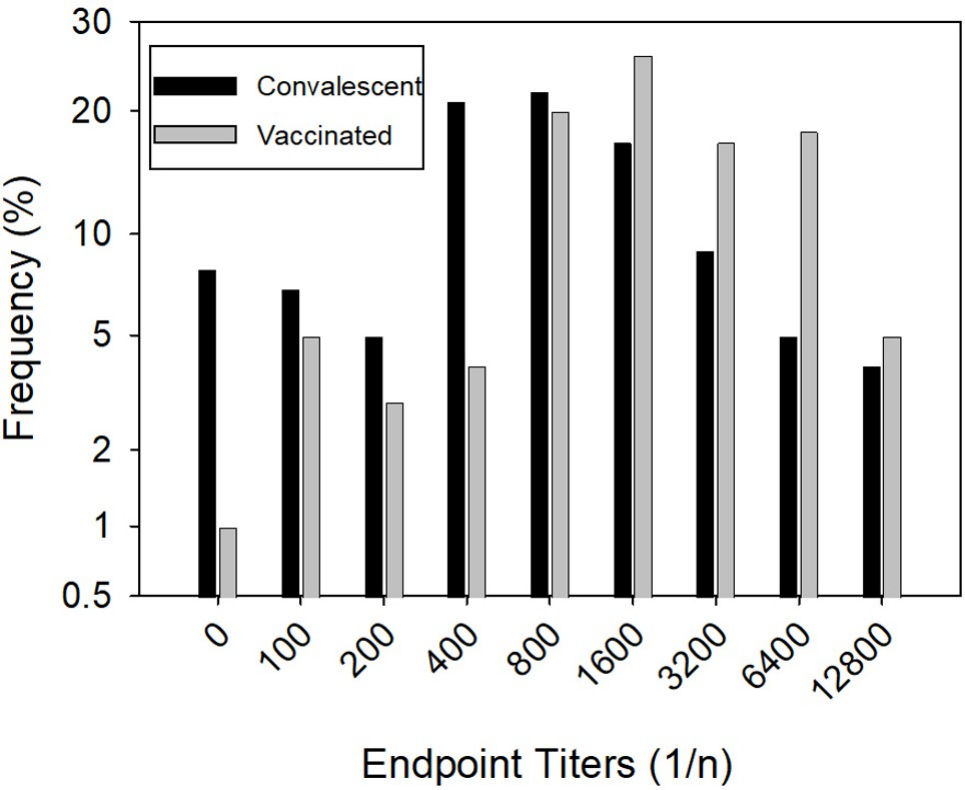
The frequencies of endpoint titers in convalescent patients and vaccinated volunteers determined by ELISA using serial dilutions. Endpoint titers were defined as the greatest dilution where a positive signal (OD_450_ ≥ 0.5) was detected.

Overall, 111/121 (91.7%) individuals were seropositive for anti-RBD IgG and had a median endpoint titer of 1:800 (Fig 2). The seven individuals without a confirmed positive COVID-19 diagnosis were all seropositive. Of the 10 individuals that were seronegative, nine were confirmed SARS-CoV-2 positive by NP-NAAT and one was diagnosed by serology. Interestingly, six of the 10 seronegative individuals did not report any symptoms associated with acute infection but had a positive diagnosis. Included in this cohort were two individuals who were vaccinated prior to study enrollment, and each had titers greater than 1:12,800.

A nonpaired analysis of 101 healthy, vaccinated adults without a documented history of prior acute COVID-19 illness had endpoint titers that were higher, a median of 1:1,600 (Fig 2), than that found for individuals with antibodies from a natural infection (P = <0.001; Mann-Whitney Rank Sum Test). These volunteers were recruited within the same northern Nevada community. Samples were collected an average of 150 days (IQR: 125–190) after vaccine regimen was complete. Despite being fully vaccinated, one individual did not have a measurable titer at the starting dilution (1:100) for the assay.

**Fig 3:**
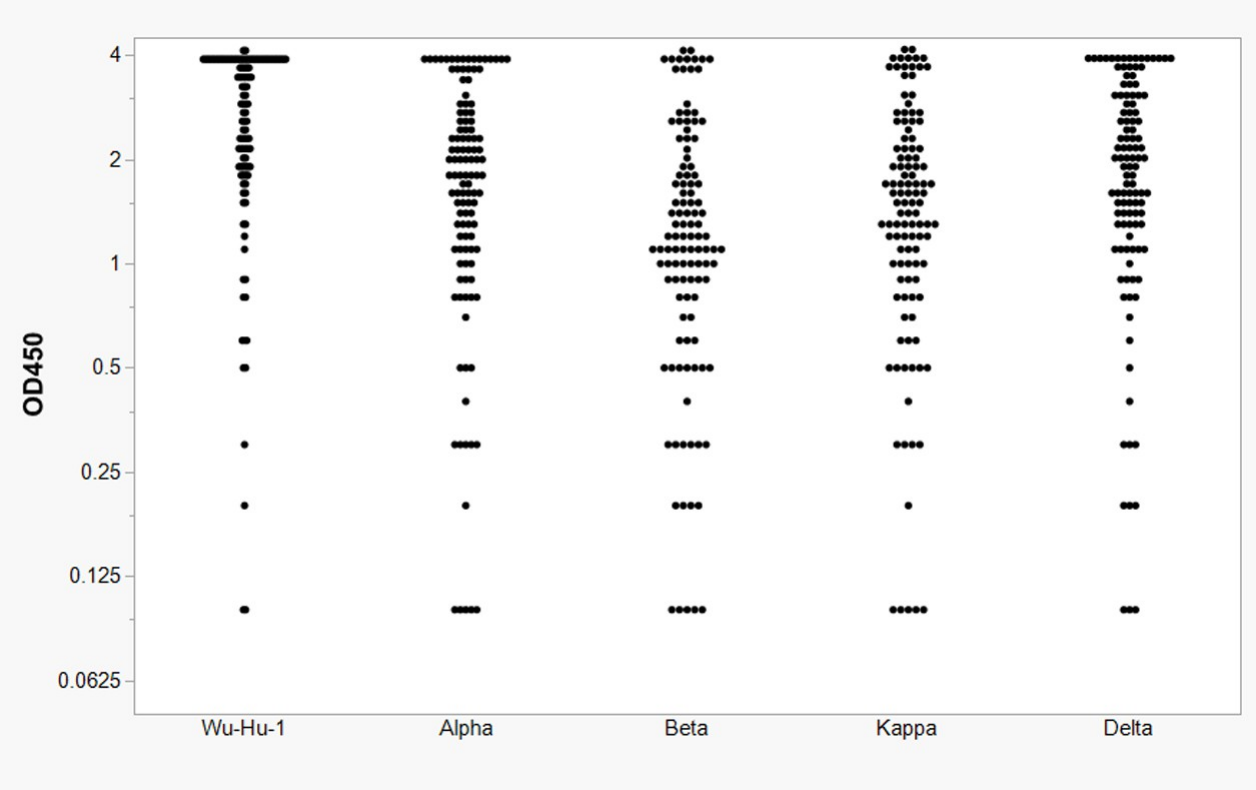
Comparative analysis of convalescent serum for anti-RBD antibodies against RBD of emerging SARS-CoV-2 variants. Serum samples were evaluated by ELISA at a single dilution (1:100) from convalescent patients against Wu-Hu-1, Alpha, Beta, Kappa, and Delta encoded RBDs.

The 121 convalescent serum samples were also screened for antibodies reactive with RBD subunits encoded by Alpha, Beta, Kappa, and Delta variants of SARS-CoV-2. A single dilution (1:100) was analyzed against recombinant RBD from each variant. Comparative analysis of the cohort’s serology against the five different variants is shown in Fig 3. The single dilution evaluation indicates a decrease in antibody levels reactive to subsequent variants compared to the original Wu-Hu-1 strain (*p*-value > 0 .00001; Wilcoxon sum rank test). The antibody response generally trended with the number of mutations in the RBD of each variant: Alpha (N501Y), Beta (K417N, E484K, N501Y), Kappa (L452R, E484Q), and Delta (L452R, T478K). Due to volume limitations, samples collected from vaccinated individuals were not screened against the other variants.

## Discussion

The present study describes the endpoint titers specific to SARS-CoV-2 (Wu-Hu-1) RBD in serum samples collected from recovered individuals enrolling in a convalescent serum screening study as well as in vaccinated individuals without prior history of COVID-19 and of the same community. Of the 121 convalescent individuals, seroprevalence was found in 91.7% of the cohort at the time of enrollment against the RBD of the Wu-Hu-1 variant. Salazar et al. indicates endpoint titers of 1:1,350 equates to viral neutralizing titers of 1:160 [9], a value set forth by the Food and Drug Administration (FDA) as a baseline for therapeutic transfusions utilized early on during the pandemic [10]. While an absolute antibody titer that is associated with protection is not fully understood this time, the present study of convalescent subjects identified 43/121 (35.2%) patient titers meeting the criterion presented by the FDA at the time of collection. Further evaluation of subsequent draws from this cohort will help elucidate the longevity of antibodies in convalescent individuals. In general, the enrolled population had mild disease which has been described to be associated with lower antibody responses [11]. Furthermore, 10 individuals (8.3%) were negative for anti-RBD titers and may represent a population that did not seroconvert to the RBD subunit or had a short-lived humoral response. As six of these individuals did not exhibit COVID-19 symptoms, it is possible they received false positive diagnoses, as seroconversion is expected by 29 days post-exposure [2]. It is possible that cell-mediated or non-RBD reactive antibodies may be important as it has been observed that exposure to related coronaviruses results in a short-term adaptive immune response leading to natural reinfections within 12 months, a trait suggested to be shared amongst coronaviruses [12].

We were also able to assess the induction of cross-variant antibodies associated with natural infection. We found less reactivity against the RBD of emerging variants compared to Wu-Hu-1, supporting the observation that natural infection may be less effective in producing cross-neutralizing antibodies to emerging variants [13].

Vaccines have been instrumental in preventing severe COVID-19 infections [14]. Among our comparative vaccinated cohort of healthy volunteers from the same community, anti-RBD antibodies were measured at an average of 150 days post-vaccination. All but one volunteer (99.0%) was seropositive. Additionally, 68/101 (67.3%) met the baseline for therapeutic transfusions. Compared to naturally infected individuals, vaccine-induced antibody responses against the Wu-Hu-1 RBD were more robust (P = <0.001; Mann-Whitney Rank Sum Test). As shown in Figs 1 and 2, there was a greater anti-RBD response in the vaccinated group versus the naturally infected group, despite a longer timeframe between immunization and sample collection. The data from this study supports both greater overall titer induction and longer duration from vaccination compared to natural infection, and confirms similar findings in other studies [15–17]. Furthermore, titers to the Wu-Hu-1 RBD confer some cross-reactive antibodies to the RBD of emerging viral isolates.

### Limitations

There are several limitations within our study design as this work was conducted during the early phases of the COVID-19 pandemic. Our study provides insight on seroconversion to the RBD subunit in convalescent individuals after an acute COVID-19 infection. Despite the non-matched nature of the vaccinated group, this study suggests that convalescent individuals have less antibodies reactive to the RBD than those induced by immunization. Limitations to this study also include a range in the collection timeline as well as minimal analysis of serum collected from the vaccinated group, and thus these data need to be interpreted with caution. Further understanding of the durability and effectiveness of natural infection and immunization are needed to develop optimal vaccine deployment strategies.

## Supporting information

S1 Table Demographic characteristics and features of convalescent patients. (PDF)
