# Supplementary material for: Serum antibody levels to SARS-CoV-2 receptor-binding domain (RBD) in convalescent patients and vaccinated individuals of northern Nevada
Source: PLoS One. 2023 Nov 2;18(11):e0288713. doi: 10.1371/journal.pone.0288713 (PMC10621914; doi:10.1371/journal.pone.0288713)
Supplement: S1 Table — (PDF) [file pone.0288713.s001.pdf]

								Wu-Hu-1		Alpha	Beta	Kappa	Delta
Sample no.	Age (yrs) at Screening	Sex	Month of Symptom Onset	COVID-19 Diagnostic Test	Result of COVID-19 Test	Sample Collection - Days From Symp. Onset	Vaccinated Prior to Screening Visit	Endpoint Titer	OD450 (1:100)	OD450 (1:100)	OD450 (1:100)	OD450 (1:100)	OD450 (1:100)
1	39	Male	2/2020	Serology (IgG)	Positive	71	No	3200	3.9	3.9	3.9	3.6	3.9
2	33	Female	3/2020	NP-NAAT	Positive	51	No	800	2.6	2.2	1.1	1.2	1.4
3	28	Female	3/2020	OP+NP-NAAT	Positive	59	No	800	3.9	2.7	2.3	2.5	2.5
4	27	Male	3/2020	NP-NAAT	Positive	58	No	100	0.8	0.7	0.3	0.3	0.2
5	38	Male	3/2020	NP-NAAT	Positive	48	No	6400	3.9	3.9	3.9	3.9	3.9
6	36	Male	3/2020	Serology (Unk)	Positive	53	No	800	2.9	1.8	1.4	1.5	1.6
7	57	Female	3/2020	NP-NAAT	Positive	46	No	400	2.3	2.6	1.1	1.2	1.5
8	51	Male	3/2020	NP-NAAT	Positive	59	No	6400	3.9	3.9	3.9	3.9	3.9
9	54	Female	3/2020	NP-NAAT	Positive	55	No	400	1.6	1.3	0.9	1.1	1.3
10	49	Female	3/2020	NP-NAAT	Positive	54	No	1600	3.9	3.6	2.7	2.9	3.9
11	60	Male	4/2020	NP-NAAT	Positive	42	No	800	2.1	2.0	1.1	1.2	1.7
12	29	Male	3/2020	NP-NAAT	Positive	61	No	400	2.2	1.6	1.0	1.0	1.0
13	30	Female	3/2020	NP-NAAT	Positive	58	No	400	1.8	1.6	1.2	1.3	1.5
14	53	Male	3/2020	NP-NAAT	Positive	58	No	1600	3.7	2.4	1.0	2.3	3.0
15	57	Female	3/2020	NP-NAAT	Positive	59	No	800	3.4	2.6	1.8	2.2	2.6
16	36	Female	3/2020	NP-NAAT	Positive	51	No	800	3.4	2.3	1.7	1.8	2.1
17	59	Female	4/2020	NP-NAAT	Positive	48	No	1600	3.9	3.4	2.3	2.6	3.5
18	52	Female	3/2020	NP-NAAT	Positive	51	No	1600	3.9	3.9	3.9	3.9	3.9
19	37	Male	3/2020	Serology (IgG)	Positive	64	No	200	1.1	1.0	0.8	0.8	0.8
20	44	Male	3/2020	Serology (IgG)	Positive	63	No	100	0.8	0.3	0.1	0.1	0.9
21	35	Male	3/2020	OP-NAAT	Positive	51	No	0	0.1	0.1	0.1	0.1	0.1
22	54	Male	3/2020	NP-NAAT	Positive	61	No	400	1.7	1.3	1.1	1.3	1.6
23	59	Female	3/2020	NP-NAAT	Positive	63	No	12800	3.9	3.9	1.7	1.9	3.9
24	45	Male	3/2020	OP+NP-NAAT	Positive	61	No	1600	3.9	3.9	2.6	3.0	3.6
25	53	Male	3/2020	NP-NAAT	Positive	63	No	400	2.1	1.4	1.0	1.2	1.5
26	60	Female	3/2020	NP-NAAT	Positive	64	No	400	1.5	1.0	0.8	1.3	1.3
27	35	Female	3/2020	NP-NAAT	Positive	52	No	3200	3.9	3.9	2.7	3.0	3.7
28	38	Male	3/2020	NP-NAAT	Positive	52	No	400	1.9	1.3	1.1	1.0	2.0
29	34	Female	4/2020	NP-NAAT	Positive	55	No	1600	3.2	2.1	1.2	1.3	3.0
30	27	Male	4/2020	NP-NAAT	Positive	41	No	800	3.6	2.4	1.8	2.1	2.3
31	33	Male	4/2020	NP-NAAT	Positive	51	No	800	3.9	1.8	1.1	1.8	2.0
32	57	Male	3/2020	NP-NAAT	Positive	57	No	800	3.7	1.7	1.0	1.9	1.8
33	53	Male	3/2020	NP-NAAT	Positive	77	No	1600	3.5	2.2	1.3	2.0	2.8
34	57	Female	4/2020	Serology (IgG)	Positive	35	No	1600	3.7	2.0	1.5	1.5	3.0
35	36	Female	3/2020	NP-NAAT	Positive	79	No	100	0.6	0.3	0.2	0.1	0.2
36	34	Male	3/2020	Serology (Unk)	Positive	74	No	400	2.7	1.2	0.9	1.2	1.3
37	22	Female	3/2020	NP-NAAT	Positive	69	No	1600	3.9	2.9	2.1	2.4	2.6
38	27	Female	3/2020	NP-NAAT	Positive	75	No	400	1.9	0.9	0.5	0.8	0.8
39	25	Female	3/2020	Serology (IgG)	Positive	82	No	100	0.9	0.5	0.3	0.5	0.5
40	34	Female	Asymptomatic	Serology (IgG)	Positive	35*	No	0	0.0	0.1	0.0	0.0	0.0
41	53	Female	3/2020	NP-NAAT	Positive	77	No	800	2.8	2.1	1.3	1.5	3.0
42	51	Female	4/2020	Serology (IgG)	Positive	72	No	3200	3.9	3.9	2.3	3.9	3.3
43	57	Female	4/2020	NP-NAAT	Positive	75	No	400	2.4	1.1	0.7	1.5	1.4
44	26	Female	5/2020	NP-NAAT	Positive	35	No	3200	3.9	3.6	3.7	3.6	3.9
45	30	Female	5/2020	Serology (IgG)	Positive	66	No	800	3.1	1.5	1.3	1.9	1.5
46	38	Female	6/2020	NP-NAAT	Positive	34	No	100	0.6	0.3	0.1	0.3	0.2
47	58	Male	Asymptomatic	NP-NAAT	Positive	56*	No	0	0.0	0.1	0.0	0.0	0.1
48	37	Female	3/2020	Serology (IgG)	Positive	123	No	800	2.2	1.4	1.1	1.7	1.4
49	44	Male	2/2020	Suspected	Suspected	171	No	3200	4.0	3.4	2.6	3.6	3.3
50	32	Female	3/2020	NP-NAAT	Positive	122	No	0	0.0	0.0	0.0	0.0	0.0
51	56	Female	1/2020	NP-NAAT	Positive	199	No	400	1.8	1.2	0.9	1.3	1.1
52	23	Female	1/2020	Suspected	Suspected	189	No	200	0.9	0.8	0.7	0.9	0.8
53	29	Male	4/2020	NP-NAAT	Positive	99	No	0	0.1	0.2	0.1	0.1	0.0
54	41	Female	3/2020	Serology (Unk)	Positive	130	No	200	1.5	1.1	0.6	1.1	1.1
55	32	Female	6/2020	NP-NAAT	Positive	45	No	100	0.6	0.3	0.2	0.5	0.3
56	45	Female	6/2020	NP-NAAT	Positive	33	No	100	0.5	0.5	0.2	0.3	0.3
57	45	Female	4/2020	NP-NAAT	Positive	103	No	800	2.7	2.3	0.4	0.5	1.9
58	52	Male	1/2020	Serology (Unk)	Positive	185	No	400	2.1	1.8	1.2	1.3	1.4
59	36	Female	6/2020	NP-NAAT	Positive	46	No	1600	3.9	3.9	2.8	2.7	3.6
60	29	Female	6/2020	NP-NAAT	Positive	51	No	200	1.2	1.1	0.5	0.5	0.7
61	22	Female	5/2020	NP-NAAT	Positive	84	No	12800	3.9	3.9	3.9	3.9	3.9
62	57	Female	6/2020	NP-NAAT	Positive	67	No	3200	3.9	3.9	2.5	2.7	3.6
63	61	Female	7/2020	Serology (IgG)	Positive	56	No	3200	3.9	3.7	2.4	2.7	3.4
64	40	Male	4/2020	NP-NAAT	Positive	142	No	400	2.0	1.1	0.5	0.8	1.2
65	24	Female	7/2020	NP-NAAT	Positive	52	No	1600	3.4	2.1	1.5	2.3	2.3
66	55	Male	4/2020	NP-NAAT	Positive	124	No	1600	3.9	2.5	2.0	2.2	2.7
67	50	Female	4/2020	NP-NAAT	Positive	124	No	400	1.9	1.5	1.1	1.2	1.6
68	67	Female	6/2020	NP-NAAT	Positive	74	No	1600	3.9	2.4	1.4	1.6	2.7
69	25	Female	6/2020	NP-NAAT	Positive	73	No	200	1.3	0.5	0.3	0.4	0.9

70	32	Male	6/2020	NP-NAAT	Positive	76	No	800	3.5	2.3	0.3	0.5	3.6
71	44	Female	Asymptomatic	NP-NAAT	Positive	112*	No	0	0.2	0.3	0.1	0.1	0.3
72	36	Male	4/2020	NP-NAAT	Positive	145	No	100	0.5	0.1	0.0	0.0	0.1
73	25	Female	Asymptomatic	NP-NAAT	Positive	48*	No	400	2.3	1.5	0.5	0.6	0.9
74	57	Male	5/2020	NP-NAAT	Positive	106	No	400	1.9	1.0	0.5	0.9	1.3
75	59	Male	Asymptomatic	NP-NAAT	Positive	81*	No	400	1.7	0.8	0.3	0.5	0.9
76	52	Male	4/2020	NP-NAAT	Positive	156	No	800	3.3	2.0	1.4	1.6	2.4
77	39	Female	6/2020	NP-NAAT	Positive	88	No	800	3.8	1.8	1.0	1.3	2.0
78	64	Female	6/6/2020	NP-NAAT	Positive	101	No	6400	3.9	3.9	3.6	3.5	3.9
79	57	Male	7/2020	NP-NAAT	Positive	69	No	1600	3.9	2.3	1.9	2.0	3.1
80	57	Female	7/2020	NP-NAAT	Positive	66	No	800	3.5	1.8	1.2	1.4	2.0
81	59	Female	7/2020	NP-NAAT	Positive	67	No	3200	3.9	1.6	1.6	1.7	3.0
82	38	Female	6/2020	NP-NAAT	Positive	103	No	400	2.3	0.8	0.6	0.8	1.6
83	51	Female	7/2020	Serology (IgG)	Positive	88	No	1600	3.9	1.6	1.0	1.7	2.4
84	51	Female	7/2020	Serology (IgG)	Positive	90	No	6400	3.9	3.6	3.9	3.6	3.9
85	46	Male	7/2020	Serology (IgG)	Positive	78	No	800	2.3	1.1	0.9	1.0	1.6
86	33	Female	8/2020	NP-NAAT	Positive	62	No	6400	3.9	3.7	3.9	3.5	3.9
87	40	Male	Missing	NP-NAAT	Positive	53*	No	800	3.5	2.0	1.1	2.0	2.3
88	29	Female	6/2020	NP-NAAT	Positive	132	No	800	2.4	0.8	0.8	1.0	1.6
89	56	Female	Asymptomatic	NP-NAAT	Positive	29*	No	0	0.0	0.1	0.0	0.0	0.0
90	58	Female	6/2020	NP-NAAT	Positive	118	No	3200	3.9	2.9	1.4	1.7	2.0
91	47	Female	Missing	NP-NAAT	Positive	125*	No	400	1.6	0.8	0.3	0.6	1.1
92	23	Female	6/2020	NP-NAAT	Positive	116	No	0	0.0	0.0	0.0	0.0	0.0
93	57	Female	6/2020	NP-NAAT	Positive	142	No	400	2.0	1.8	1.2	1.6	1.9
94	55	Female	7/2020	NP-NAAT	Positive	82	No	3200	3.9	3.7	0.5	0.2	0.6
95	53	Female	Asymptomatic	NP-NAAT	Positive	158*	No	0	0.0	0.0	0.0	0.0	0.0
96	61	Female	8/2020	NP-NAAT	Positive	77	No	800	3.0	2.0	1.5	1.7	2.2
97	37	Male	6/2020	NP-NAAT	Positive	139	No	100	0.6	0.4	0.2	0.3	0.4
98	41	Female	6/2020	NP-NAAT	Positive	120	No	800	2.8	2.0	1.7	1.7	1.9
99	26	Female	Asymptomatic	NP-NAAT	Positive	134*	No	0	0.0	0.0	0.0	0.0	0.0
100	59	Male	6/2020	NP-NAAT	Positive	126	No	400	1.9	1.2	1.1	1.1	1.5
101	49	Female	6/2020	NP-NAAT	Positive	127	No	800	2.5	1.6	1.3	1.4	1.8
102	60	Female	9/2020	NP-NAAT	Positive	41	No	200	1.3	0.9	0.6	0.7	1.1
103	50	Male	7/2020	NP-NAAT	Positive	101	No	12800	3.9	3.9	3.6	3.6	3.9
104	53	Male	6/2020	Serology (Unk)	Positive	139	No	800	2.8	2.3	1.8	1.9	2.3
105	65	Male	9/2020	NP-NAAT	Positive	56	No	400	1.9	1.5	0.9	0.7	1.1
106	54	Female	6/2020	NP-NAAT	Positive	138	No	400	1.8	1.4	1.0	1.0	1.4
107	48	Male	Asymptomatic	NP-NAAT	Positive	136*	No	1600	3.7	2.7	2.5	2.5	2.9
108	48	Female	Missing	NP-NAAT	Positive	71*	No	6400	3.9	3.9	4.0	3.7	3.9
109	32	Female	9/2020	NP-NAAT	Positive	72	No	800	2.5	1.6	1.0	1.9	2.2
110	43	Female	7/2020	NP-NAAT	Positive	150	No	400	2.2	1.8	1.2	1.3	1.6
111	50	Male	7/2020	NP-NAAT	Positive	157	No	1600	3.9	2.8	2.7	2.7	3.3
112	63	Female	7/2020	NP-NAAT	Positive	169	No	800	3.2	2.3	1.9	2.1	2.6
113	24	Male	10/2020	NP-NAAT	Positive	83	Yes	12800	3.9	3.9	3.7	4.0	3.9
114	71	Female	10/2020	Serology (Unk)	Positive	76	No	1600	3.8	2.7	1.5	1.6	2.1
115	58	Male	9/2020	NP-NAAT	Positive	123	No	1600	3.9	2.0	1.7	1.6	2.0
116	38	Female	11/2020	NP-NAAT	Positive	59	No	1600	3.9	2.1	1.6	1.7	2.2
117	40	Female	11/2020	NP-NAAT	Positive	61	No	800	2.3	1.3	0.9	0.9	1.3
118	59	Female	8/2020	NP-NAAT	Positive	168	No	400	1.8	0.9	0.5	0.6	1.1
119	40	Female	11/2020	NP-NAAT	Positive	87	Yes	12800	4.0	3.9	4.0	4.0	3.9
120	20	Female	9/2020	NP-NAAT	Positive	137	No	1600	3.9	1.7	1.4	1.4	1.7
121	56	Female	9/2020	NP-NAAT	Positive	134	No	3200	3.9	3.0	2.5	2.6	2.7

*Days from postive test result
